# Deoxyribonuclease 1 reduces pathogenic effects of cigarette smoke exposure in the lung

**DOI:** 10.1038/s41598-017-12474-5

**Published:** 2017-09-21

**Authors:** Paul T. King, Roleen Sharma, Kim M. O’Sullivan, Judy Callaghan, Lovisa Dousha, Belinda Thomas, Saleela Ruwanpura, Steven Lim, Michael W. Farmer, Barton R. Jennings, Michaela Finsterbusch, Gavin Brooks, Stavros Selemidis, Gary P. Anderson, Stephen R. Holdsworth, Philip G. Bardin

**Affiliations:** 1 Monash Lung and Sleep, Monash Medical Centre, Melbourne, Australia; 2 Centre for Inflammatory Diseases, Monash University Department of Medicine, Monash Medical Centre, Melbourne, Australia; 3 Monash Micro Imaging, Monash University, Melbourne, Australia; 4 Department of Pharmacology, University of Melbourne, Melbourne, Australia; 5 Hudson Institute of Medical Research, Melbourne, Australia; 6 Program in Chronic Infectious and Inflammatory Diseases, School of Health and Biomedical Sciences, RMIT University, Melbourne, Australia

## Abstract

Our aim was to investigate if deoxyribonuclease (DNase) 1 is a potential therapeutic agent to reduce pathogenic effects of cigarette smoke exposure in the lung. Cigarette smoke causes protease imbalance with excess production of proteases, which is a key process in the pathogenesis of emphysema. The mechanisms responsible for this effect are not well-defined. Our studies demonstrate both *in vitro* and *in vivo* that cigarette smoke significantly increases the expression of neutrophil and macrophage extracellular traps with coexpression of the pathogenic proteases, neutrophil elastase and matrix metalloproteinases 9 and 12. This response to cigarette smoke was significantly reduced by the addition of DNase 1, which also significantly decreased macrophage numbers and lung proteolysis. DNase 1, a treatment currently in clinical use, can diminish the pathogenic effects of cigarette smoke.

## Introduction

Cigarette smoke exposure induces chronic inflammation in the lung, as well as causing widespread extrapulmonary manifestations. Lung inflammation is the primary cause of emphysema and chronic obstructive pulmonary disease (COPD) and persists after smoking cessation^1,2^. Currently available anti-inflammatory therapy for emphysema and chronic obstructive pulmonary disease (COPD) is only partially effective and does not reverse the underlying processes^3^.

Proteases are produced in the lung and have an important role in tissue remodelling. Protease imbalance is considered to be a cardinal pathway in the pathogenesis of emphysema and COPD, as well other chronic inflammatory lung diseases including cystic fibrosis (CF) and bronchiectasis^4–7^. Macrophages express proteases, including matrix metalloproteinase (MMP) 9 and MMP12 and neutrophils express neutrophil elastase (NE). The excessive and sustained production of proteases and/or deficiency of inhibitors (e.g. alpha-1 antitrypsin)^4,8^ results in protease imbalance and this may mediate the development of lung pathology^4–7,9,10^. Cigarette smoke is a stimulus known to induce protease expression but the mechanisms involved have not been clearly defined.

One mechanism linked to protease expression and inflammation is extracellular trap formation. Extracellular traps are produced by neutrophils (NETs) and are composed of extracellular processed chromatin bound to granular and selected proteins (e.g. granule proteases). NETs are most commonly expressed as an inflammatory response to infection (especially bacteria) and have an important role in host defence. The production of reactive oxygen species (ROS) has a primary role in initiating their generation^11,12^. The colocalisation of proteases with chromatin, citrullination of histone (H3Cit) and the production of peptidylarginase deiminase (PAD) 4 are specific features of NETosis, which distinguish it from other cellular pathways such as apoptosis^13–17^. Recently it has been recognised that other leukocytes may also produce extracellular traps including macrophages (METs)^12,18,19^. METs are not a well-defined pathology in the literature but one characteristic feature of extracellular traps is that production is dismantled extracellularly by the addition of deoxyribonuclease (DNase) 1. DNase I degrades extracellular chromatin and is currently used as a clinical treatment to improve sputum clearance by degrading host and bacterial DNA in the airways of patients with cystic fibrosis (CF).

We hypothesized that cigarette smoke induces protease expression via macrophage and neutrophil extracellular trap formation. We also postulated that the expression of NETs and METs and consequent pathogenic effects would be reduced by the addition of DNase 1.

## Results

### Cigarette smoke induces the expression of METs

**Figure 1:**
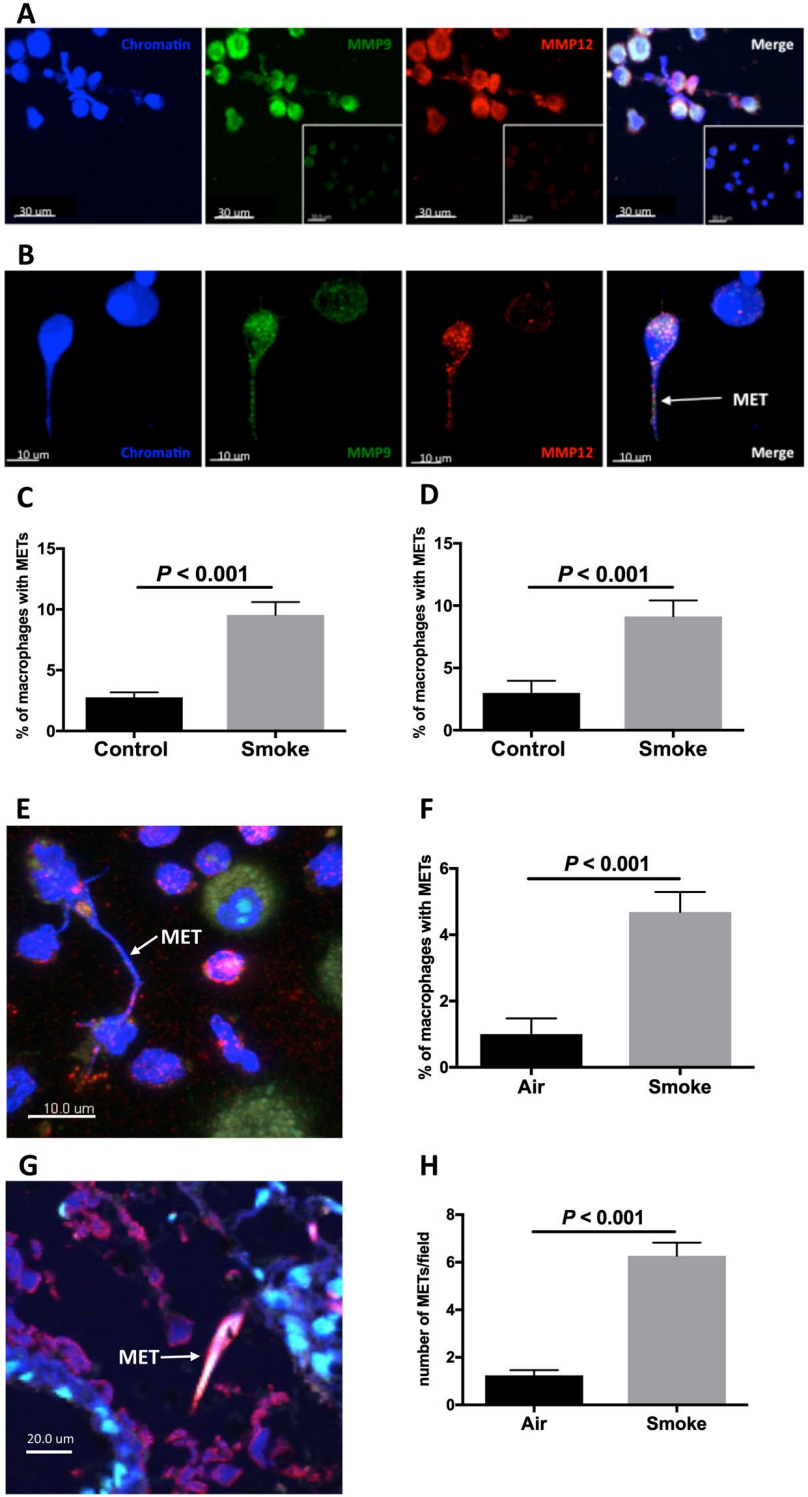
Cigarette smoke exposure induces formation of macrophage extracellular traps. Cigarette smoke extract (CSE) induces macrophage extracellular trap (MET) formation in human bronchoalveolar (BAL) macrophages *in vitro* (**A**–**D**). (**A**) Represenative image shows MET formation as characterised by extracellular chromatin, matrix metalloproteinase (MMP) 9, MMP12 and merged image; insert shows isotype control. Scale bars = 30 μm. (**B**) High resolution image of MET. Scale bars = 10 μm. (**C**) MET expression as characterised by extracellular chromatin, MMP9 and 12 (*n* = 20 individuals). (**D**) MET expression as characterised by extracellular chromatin and citrullinated histone (H3Cit), (*n* = 13). Exposure to cigarette smoke induces MET expression in murine lung *in vivo* (**E**–**H**). (**E**) Image shows MET formation by BAL macrophages. Scale bar = 10 μm. (**F**) MET expression by murine BAL macrophages (*n* = 13). (**G**) Image of MET in murine lung tissue. Scale bar = 20 μm. (**H**) MET expression by lung tissue macrophages (*n* = 10). Results are shown as air (or control) and smoke exposure (*in vitro* CSE or *in vivo* cigarette smoke exposure).

We first assessed whether cigarette smoke extract (CSE) induced MET expression *in vitro*. Alveolar macrophages were obtained by bronchoalveolar lavage (BAL) from patients undergoing bronchoscopy (Table S1 in the Supplementary Information). Alveolar macrophages were exposed to CSE for one hour before being fixed and phenotyped with specific markers and analysed for MET expression by confocal microscopy (Table S2). MET expression was measured by the extracellular production of chromatin and colocalisation with the proteases MMP9 and MMP12 (representative images are shown in Fig. 1A and B). CSE induced an increase in MET expression (*P* < 0.001, Fig. 1C). To further characterise METs we used an additional method to identify MET formation by demonstrating the coexpression of H3Cit with extracellular chromatin. CSE upregulated MET formation (*P* < 0.001, Figs 1D and S1 in the online data supplement). We further characterised MET formation by demonstrating colocalisation of chromatin/H3Cit with either MMP9 or PAD2 (Figures S1 and S2 in the Supplementary Information).

In a subgroup analysis of the patients from whom BAL was obtained, subjects with a significant smoking history (>10 pack-years) and emphysema were compared to subjects without a significant smoking history and no known lung disease. Both groups demonstrated upregulation of MET production (*P* < 0.05, Figure S3).

To examine MET expression *in vivo*, mice were exposed to cigarette smoke for 4 days and then BAL macrophages were obtained. METs were defined by the colocalisation of extracellular chromatin, MMP9 and H3Cit. Cigarette smoke increased the production of METs from BAL macrophages *in vivo* (*P* < 0.001, Figs 1E,F and S4). We also measured the production of METs in the lungs of smoke-exposed mice and detected an increase in MET expression (*P* < 0.001, Figs 1G,H and S5). Thus both *in vitro* and *in vivo*, cigarette smoke induces MET production with protease coexpression.

### Cigarette smoke induces the expression of NETs

**Figure 2:**
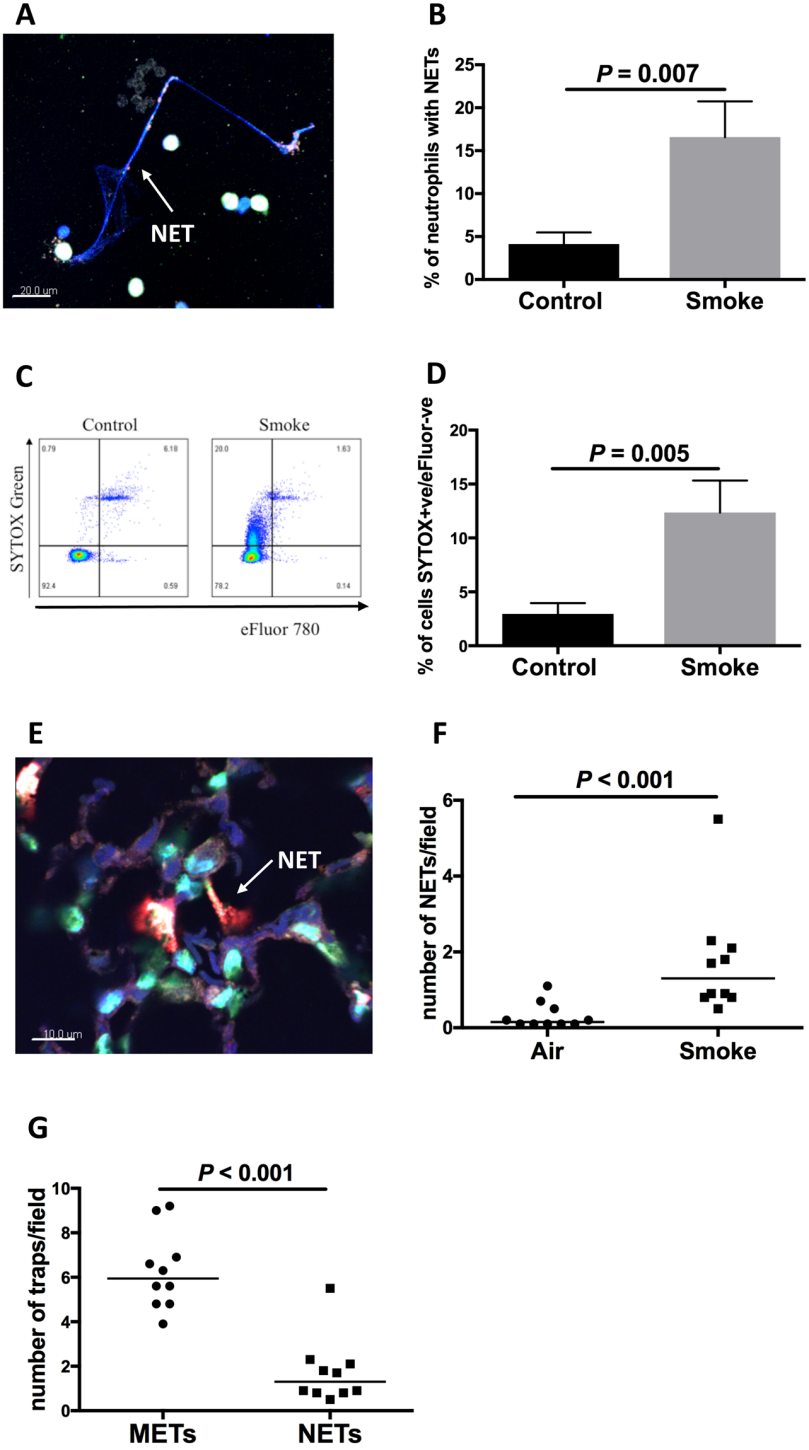
Cigarette smoke exposure induces formation of neutrophil extracellular traps. CSE induces neutrophil extracellular trap (NET) formation in human peripheral blood neutrophils *in vitro* (**A**–**D**). (**A**) Representative image shows NET formation as characterised by staining for extracellular chromatin, H3Cit, peptidylarginase deiminase (PAD) 4 and neutrophil elastase (NE). Scale bar = 20μm. (**B**) NET expression in blood neutrophils (*n* = 8 individuals). (**C**) A flow cytometry method was also developed to measure NET production, with SYTOX green staining for extracellular chromatin production and eFluor as a marker of cell death. Dot plot shows NET expression as characterised by positive staining for SYTOX green and negative for eFlour. (**D**) NET expression by neutrophils demonstrated using flow cytometry (*n* = 8). Exposure to cigarette smoke induces NET expression in mice *in vivo* (**E**,**F**). (**E**) Image shows NET formation in lung tissue. Scale bar = 10μm. (**F**) NET expression in lung tissue (*n* = 10). (**G**) In lung tissue, cigarette smoke induced a higher number of METs than NETs.

We used an established method to define NETs with coexpression of extracellular chromatin, the protease NE, H3Cit and PAD4 (PAD4 is present in neutrophils,whilst PAD2 is more prominent in macrophages), (representative images are shown in Figs 2A and S6). CSE significantly upregulated NET expression i*n vitro* in peripheral blood neutrophils obtained from healthy human controls (*P* = 0.007, Fig. 2B). We used blood neutrophils because numbers of neutrophils in BAL were generally too low to examine expression of NETs.

Another method that has been used to define the expression of NETs is using the non-cell permeable DNA dye SYTOX green^11^. We developed a flow cytometry method using SYTOX green and demonstrated that CSE upregulated NET expression in human neutrophils (*P* = 0.005, Fig. 2C and D). We were not able to use this SYTOX method successfully for the demonstration of METs as the macrophages were adherent after stimulation and could not be removed.

We also confirmed upregulated NET expression in lung tissue from mice that had been exposed to cigarette smoke (*P* < 0.001, Fig. 2E,F and Figure S7). The numbers of METs were approximately 5-fold higher than the numbers of NETs in the lung tissue in this model (Fig. 2G).

### Comparison of METs and NETs

There is minimal published literature that compares METs and NETs. Our previous published data showed that the common lung bacterium nontypeable *Haemophilus influenzae* induced a higher percentage of neutrophils to produce extracellular traps when compared to macrophages^19^. In the current study there were notable differences in morphology between NETs and METs. The length of extracellular traps in the human samples was measured showing that NETs had longer extensions of extracellular chromatin (*P* = 0.029, Figure S8A). An example of this can be seen when comparing the image of a NET in Fig. 2A with a MET in Fig. 1B. Neutrophils and macrophages have different roles in host defence and this may be reflected in the particular characteristics of their respective extracellular traps.

The published literature highlights the critical role of the oxidative burst with ROS production in the generation of NETs^11–13^. Cigarette smoke causes oxidative stress; it contains large numbers of oxygen radicals and also induces an inflammatory response by phagocytes with subsequent ROS production. CSE increased intracellular ROS levels in human macrophages (*P* = 0.041, Figure S9A) and neutrophils (*P* = 0.031, Figure S9B), and this process is likely to have a key role in the development of extracellular traps. ROS production was higher in CSE exposed neutrophils than in macrophages (*P* = 0.043, Figure S8B).

### DNase 1 reduces the expression of extracellular traps, lung macrophage numbers and lung proteolysis

**Figure 3:**
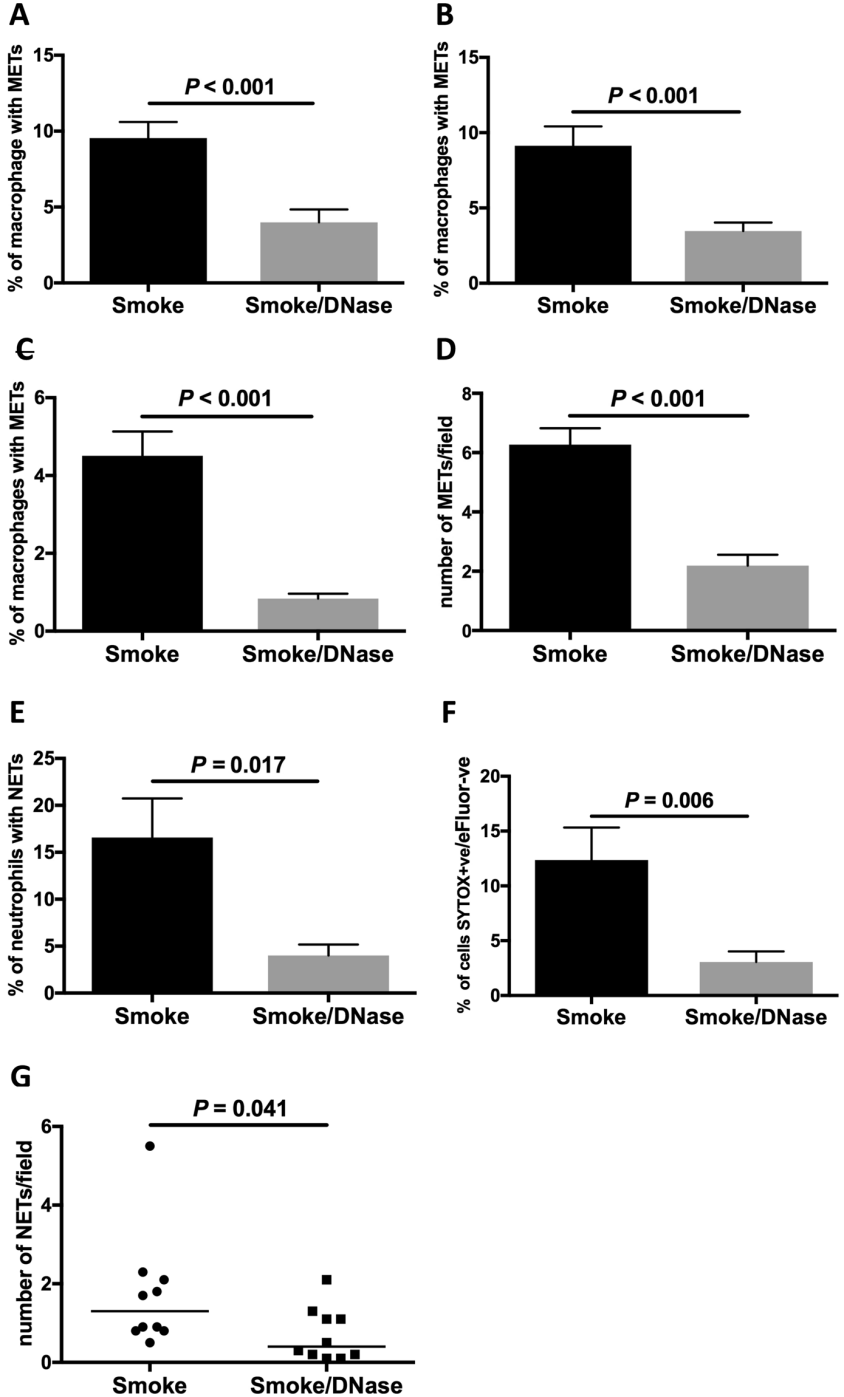
Deoxyribonculease 1 disrupts macrophage and neutrophil extracellular trap formation. The addition of deoxyribonuclease (DNase) 1 reduces the expression of cigarette smoke-induced METs. MET expression *in vitro* (**A**,**B**): (**A**) CSE-induced human BAL METs (chromatin/MMP9/12), (*n* = 20), and (**B**) CSE-induced human BAL METs (chromatin/H3Cit), (*n* = 14). MET expression *in vivo* in murine lung (**C**,**D**): (**C**) BAL METs (*n* = 10), and (**D**) lung tissue METs (*n* = 10). The addition of DNase 1 reduces the expression of cigarette smoke-induced NETs (**E**,**F**): (**E**) CSE-induced human blood NETs (*n* = 8), and (**F**) NETs as characterised using flow cytometry (*n* = 8). NET expression *in vivo* in mice: (**G**) lung tissue NETs (*n* = 10). Results are shown as smoke exposure (*in vitro* CSE or *in vivo* cigarette smoke exposure) and the effect of the addition of DNase 1 on this exposure.

DNase 1 has been shown to dismantle extracellular trap formation^11,12^. The addition of DNase 1 decreased cigarette smoke-induced MET expression *in vitro* (*P* < 0.001, Fig. 3A and B) and *in vivo* (*P* < 0.001, Fig. 3C and D). DNase 1 also decreased cigarette smoke-induced NET expression, both *in vitro* (*P* = 0.017, Fig. 3E), (P = 0.006, Fig. 3F) and *in vivo* (*P* = 0.041, Fig. 3G).

**Figure 4:**
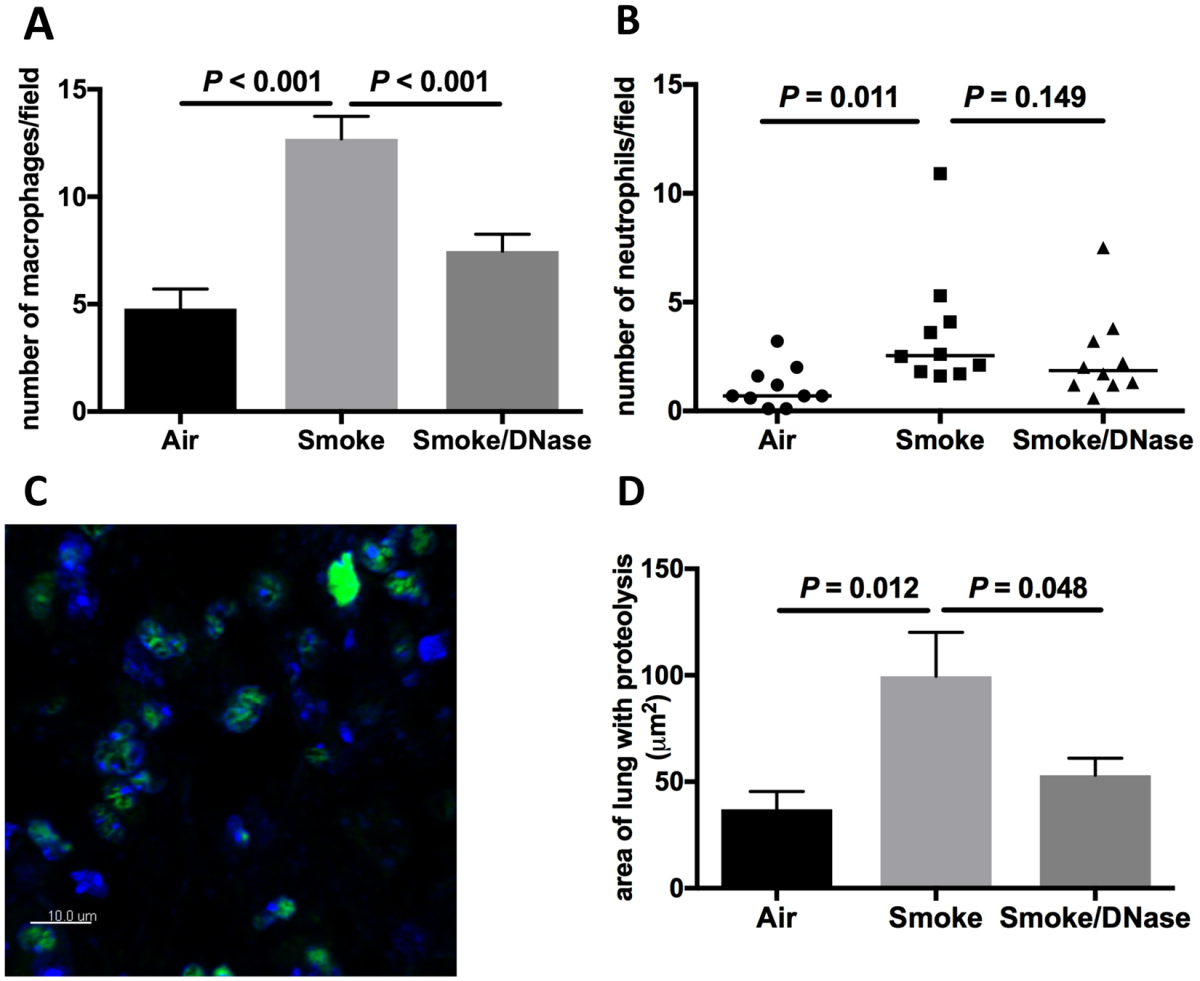
Deoxyribonculease 1 reduces lung macrophages and areas of proteolysis. Cigarette smoke increases numbers of macrophages and neutrophils and the area of the lung with proteolysis in a murine model *in vivo*. The addition of DNase 1 reduces macrophage numbers and lung proteolysis. (**A**) Number of macrophages in the lung (*n* = 10). (**B**) Number of neutrophils in the lung (*n* = 10). The area of lung demonstrating area of proteolytic change was assessed using *in situ* zymography. (**C**) Shows staining for chromatin (blue) and staining for proteolysis (green). (**D**) Area of lung with proteolysis (*n* = 10).

Cigarette smoke increased the numbers of macrophages in mouse lung tissue (*P* < 0.001) and the addition of DNase reduced numbers of macrophages (*P* < 0.001, Fig. 4A). Cigarette smoke increased numbers of neutrophils in lung tissue (*P* = 0.011) but DNase did not appear to reduce neutrophils (Fig. 4B).

To provide further confirmation of the specific activties of DNase, we included studies of a vehicle control (with no DNase) in smoke-exposed mice. The vehicle control did not cause a significant reduction in lung METs, NETs or macrophages (Figure S10).

Zymography can be used to define the prescence of functionally activated proteases. *In situ* zymography enables the localisation and quantitation of matrix-degrading protease activity in histological sections^20^. We used *in situ* zymography to demonstrate that cigarette smoke in mouse lung tissue increased the area of lung with proteolysis (*P* = 0.012) and that this was reduced by the addition of DNase (*P* = 0.048, Figs 4C,D and S11). The dose of DNase we used for these experiments did not have a major effect on cell viabilitty (Figure S12)

## Discussion

We have shown both *in vitro* and *in vivo*, that cigarette smoke is a potent inducer of extracellular trap formation by lung macrophages and neutrophils. METs and NETs express proteases including macrophage metalloproteinases 9 and 12 and neutrophil elastase. Importantly, these proteases have been shown to have a key role in the development of protease imbalance, lung inflammation and lung pathology in COPD. Extracellular trap formation induced by cigarette smoke was effectively dismantled by DNase 1.

COPD is characterised by two primary pathogenic processes that generally occur together; emphysema and chronic bronchitis. Proteases have an important role in the digestion of the extracellular matrix and contribute to remodelling of lung tissue. Protease imbalance has been most clearly demonstrated in the pathogenesis of emphysema. It also has a key role in other inflammatory lung conditions including cystic fibrosis and bronchiectasis^4–7^. This imbalance is characterised by the overproduction of pathogenic lung proteases and/or deficiency of antiproteases (e.g. alpha-1 antitrypsin). Key proteases that have been identified in this context include neutrophil elastase and MMP 1, 9 and 12. MMP12 knockout mice are protected from emphysema^10^ and patients with polymorphisms of MMP9 have increased risk of emphysema^21^. Proteases are present inside immune cells such as macrophages and neutrophils. Smoking has been shown to induce extracellular expression of active proteases in the lung but the mechanisms involved are poorly understood.

Neutrophil extracellular traps are composed of extracellular processed chromatin bound to granular and selected proteins^11,12,22^. NETs are primarily induced in response to infection (predominantly bacteria) and are generally pro-inflammatory. In addition to their role in protection against infection, NETs have been described in noninfectious disease such as autoimmune conditions (e.g. systemic lupus erythematous), in thrombosis and in response to the mitogen phorbol myristate acetate^11,12,23^. There have been several recent reports which have described NETs occuring in patients with COPD^24–26^; with traps being demonstrated both in stable and exacerbated COPD and correlating with inflammatory markers. Two recent studies have described that nicotine induces NETosis *in vitro*. Both studies demonstrated in healthy human controls that nicotine acts on nicotinic aceltylcholine receptors on peripheral blood neutrophils to induce NET formation^27,28^. Nicotinic receptors do not have a clearly defined direct role on lung phagocyte function in the lung. The nicotinic pathway acts in the nervous system (and is responsible for the addictive effect of nicotine). The nicotinic pathway acts on the lung via the vagus nerve having an anti-inflammatory effect with downregulation of inflammatory cytokine production^29^. There are no reports to our knowledge describing an association between smoking and extracellular trap formation in the lung and the potential influence of DNase 1 on this process.

The production of extracellular traps also occurs in other cell types including macrophages^12,18^. Macrophages have similar properties to neutrophils with production of ROS in response to infection and the production of proteases. We have previously described MET formation in human BAL macrophages after exposure to the common respiratory bacterium, nontypeable *Haemophilus influenzae* (NTHi)^19^. In the current study we have demonstrated that cigarette smoke induced both NET and MET formation with pathogenic protease coexpression. The production of METs was approximately 5-fold higher than NET production in response to cigarette smoke.

Although the primary aim of this study was to investigate if cigarette smoke induced the formation of extracellular traps, we also found that subjects without a significant smoking history (and no definable lung disease) and those with a significant smoking history (and evidence of emphysema) had an increase in MET expression on exposure to cigarette smoke. The current studies were not powered to ascertain if subjects who smoke and have evidence of emphysema have higher MET expression than non-smoking subjects. Several factors make this a complex question. In addition to extracellular trap formation there are other factors which may alter protease expression, the most well-known being anti-proteases such as alpha-1 antitrypsin. Also, patients with emphysema are a heterogeneous group and COPD occurs in only a proportion of smokers. Clearly, further investigation in well-characterised patients with COPD and emphysema would be of interest.

An important feature of extracellular traps is that their expression is degraded by the addition of DNase 1. We used DNase 1 which breaks down extracellular DNA, and found that DNase 1 was highly effective in degrading extracellular trap formation both *in vitro* and *in vivo*. In addition, DNase 1 reduced the numbers of macrophages in the lung tissue induced by cigarette exposure. Proteases induce chemotaxis^30,31^ and the reduction of lung macrophages is consistent with this action. DNase 1 has been shown to reduce lung neutrophil numbers in a mouse model of pancreatitis, but the mechanism is not defined^32^. Using *in situ* zymography we found that DNase 1 reduced the area of lung with proteolysis. We speculate that this effect may be the result of a combination of inhibition of METs/NETs and decreased macrophage numbers - and potentially also neutrophil numbers. In a mouse model of sickle cell disease, parenterally administered DNase 1 reduced mortality and the primary mechanism was via dimantling of pulmonary NETs^33^.

A striking feature of COPD is that inflammation continues after smoking cessation^1,2^. The factors that drive this inflammation after smoking cessation have not been well defined. Treatment of COPD is only partially effective; no currently available pharmaceutical therapy reduces mortality or reverses the inflammatory process that drives the condition^3^. Taken together our findings indicate that targetting lung MET/NET expression induced by cigarette smoke by treatment with DNase may potentially have key anti-inflammatory benefits. This can occur via reductions in lung protease expression and macrophage infiltration.

We identified limitations of this study. In patients with cystic fibrosis (CF), DNase 1 (dornase alfa) is given by a nebulized route. In this study we gave DNase 1 by a systemic route as this is standard method that has been used previously and has been demonstrated to have effects on lung NET expression. The parenteral route is the standard method for administering high-dose medication to the lung in patients (e.g. antibiotics and corticosteroids). Adminstering the DNase by a nasal route would have more variability in the dose given to the mice. Emphysema and COPD are heterogeneous conditions and from this study we are not able to determine, which subgroups of patients are most likely to derive potential benefit from the adminstration of DNase 1. Finally, we only demonstrated the effect of DNase in short-term smoke exposure models. It will be important to expand this research to assess the effect of DNase 1 in chronic smoke-exposure murine models.

Nebulized DNase 1 is available commercially in the form of dornase alfa which has been used to improve sputum clearance in patients with CF by breaking down extracellular bacterial DNA^34^. The long-term use of dornase alfa has been demonstrated to be safe, economical and associated with improved clinical outcomes in patients with CF^34^. However a previous trial in patients with non-CF bronchiectasis did not demonstrate clinical benefit^35^. Based on our findings we believe that the use of DNase 1 may have a potential role in the treatment of smoking-induced protease imbalance and macrophage infiltration to prevent tissue damage. This needs to balanced against the inhibition of entracellular trap formation may cause increased risk of respiratory infection (particularly in patients who have microbial airway colonisation). DNase 1 is a potentially powerful immunomodulatory agent for the treatment of patients with COPD but may be most effective for specific indications; perhaps as an addition to antibiotics and corticosteroids for the management of exacerbations or for those patients who have rapidly progressive emphysema.

In summary, we have demonstrated that cigarette smoke induces pulmonary neutrophil and macrophage extracellular trap formation with attendant protease expression. This may be an important mechanism for the development of protease imbalance, a key factor in the pathogenesis of emphysema/COPD and other inflammatory lung conditions. The addition of DNase 1 decreased cigarette smoke-induced MET/NET expression as well as decreasing macrophage numbers and proteolysis in the lung. This proof-of-concept study implies a potential new therapeutic approach in COPD.

## Methods

### Study subjects and animals

Human studies were approved by the Ethics Committee of Monash Medical Centre (MMC)/Monash Health, Melbourne, Australia. Informed consent was obtained from all subjects. The methods were carried out in accordance with the relevant guidelines and regulations. Murine experiments were approved by the Anatomy and Neuroscience, Pathology, Pharmacology and Physiology AEC Ethics Committee of the University of Melbourne and experiments were conducted as specified (in accordance with) by the Ethics Committee.

We studied patients who were referred to have bronchoscopy at MMC/Monash Health. Bronchoalveolar lavage (BAL) was done to obtain lung macrophages. Neutrophils were also obtained from peripheral blood of healthy control subjects using density gradient isolation.

### Study design and methods

To study the effect of cigarette smoke exposure *in vivo*, an established mouse model was used^36^. In this whole-body smoke exposure model, mice were exposed to cigarette smoke three times a day for 4 days. Sham-exposed mice underwent the same procedure, but without cigarette smoke. On the 4^th^ day, BAL samples were taken and for analysis of whole-lung samples, lung tissue was inflated and fixed. To assess the effect of DNase, DNase 1 was administered twice a day intraperitoneally at a dose of 1 × 10^4^ international units per kg.

An established method was used for the generation of cigarette smoke extract for *in vitro* experiments^37^. For confocal microscopy cells (macrophages and neutrophils) were seeded onto cover slips. Cells were stimulated with CSE or CSE/DNase for one hour in culture medium (dose of DNase 2 μg/ml).

We used established flow cytometry methods to measure the production of ROS from neutrophils and macrophages by cleavage of the dye dihydrorhodamine 123 (DHR)^19^. To measure the expression of NETs, cells were labelled with the cell impermeable marker SYTOX green and fixable viability dye eFluor 780.

Established methods were used^11,19,38^. To define the prescence of extracellular traps, fixed cells/tissue were permeabilised and then labelled with primary and secondary antibodies. Multiple different protocols were used to define METs and NETs. Images were captured using invert laser-scanning microscopy. Analysis was performed using IMARIS imaging analysis software. The length of extracellular traps was measured using Image J. In lung tissue, macrophages and neutrophils were defined by the number of cells per high-powered field with staining for the specific markers of F4/80 (macrophages) and NE (neutrophils). *In situ* zymography was performed using nonfixed, noninflated lung tissue samples, which were immersed in a highly-quenched fluorescent substrate solution to define areas of the lung with proteolysis.

### Statistical analysis

Comparisons between control and cigarette smoke-exposed groups were done using paired or unpaired testing with parametric or non-parametric methods as appropriate. Parametric results are represented graphically by mean and standard error of the mean (SEM), whilst non-parametric results are represented graphically by medians. Statistical analysis was performed using Prism 6 software.

More detailed methods are outlined in the Supplementary Infomation.

### Data availability

The datasets generated during and/or analysed during the current study are available from the corresponding author on reasonable request and/or; data generated or analysed during this study are included in this published article (and its Supplementary Information files).

## Electronic supplementary material

Supplementary information
