# Supplementary material for: Deoxyribonuclease 1 reduces pathogenic effects of cigarette smoke exposure in the lung
Source: Sci Rep. 2017 Sep 21;7:12128. doi: 10.1038/s41598-017-12474-5 (PMC5608940; doi:10.1038/s41598-017-12474-5)
Supplement: Supplementary file 1 — Supplementary information [file 41598_2017_12474_MOESM1_ESM.pdf]

Supplementary Information

Deoxyribonuclease 1 reduces pathogenic effects of cigarette smoke exposure in the lung

Authors: Paul.T. King[1, 2 * †], Roleen. Sharma[1, 2 †], Kim. M. O'Sullivan[2], Judy. Callaghan[3], Lovisa. Dousha[4], Belinda. Thomas[1, 5], Saleela. Ruwanpura[5], Steven. Lim[2], Michael. W Farmer[1], Barton. R. Jennings[1], Michaela. Finsterbusch[2], Gavin. Brooks[5], Stavros. Selemidis[6], Gary. P. Anderson[4], Stephen. R. Holdsworth[2], Philip. G. Bardin[1, 5]

Affiliations:

1. Monash Lung and Sleep, Monash Medical Centre, Melbourne, Australia.
2. Centre for Inflammatory Diseases, Monash University Department of Medicine, Monash Medical Centre, Melbourne, Australia.
3. Monash Micro Imaging, Monash University, Melbourne, Australia.
4. Department of Pharmacology, University of Melbourne, Melbourne, Australia.
5. Hudson Institute of Medical Research, Melbourne, Australia.
6. Program in Chronic Infectious and Inflammatory Diseases, School of Health and Biomedical Sciences, RMIT University, Melbourne, Australia.

[†] These authors contributed equally to this work.

Materials and Methods

Study design

Human studies were approved by the Ethics Committee of Monash Medical Centre (MMC)/Monash Health, Melbourne, Australia. Informed consent was obtained from all subjects after the nature and possible consequences of the studies were explained. Murine experiments were approved by the Anatomy and Neuroscience, Pathology, Pharmacology and Physiology AEC Ethics Committee of the University of Melbourne and experiments were conducted as specified by the Ethics Committee. We determined group sizes based on our previously published work and preliminary data. Data inclusion and exclusion criteria were established prior to the commencement of the study and are listed below. There was no randomisation in the experiments and we did not exclude any samples. The investigators were not blinded to allocation of samples/mice or analysis of the results.

Bronchoalveolar lavage (BAL) patients

Patients were referred to have a bronchoscopy at Monash Medical Centre (MMC)/Monash Health. Of the 34 patients, 32 of the subjects were referred to MMC/Monash Health after assessment by an affiliated respiratory physician, whilst two other subjects were referred from other medical institutions. Subjects had lung function testing performed using GOLD guidelines[1] with the best post-bronchodilator response used for results and lung diffusing capacity performed. Two of the subjects were inpatients and the rest were outpatients.

Twelve subjects of the total cohort had a significant smoking history (> than 10 pack-years), but only three subjects of the total group were active smokers at the time of bronchoscopy. Of the 12 subjects, six had had computed tomography scans which were reported by a consultant

radiologist to show emphysema. In the other 6 subjects, all had low lung diffusing capacity on lung function testing (without any other detectable lung disease that would explain a low lung diffusing capacity). A low lung diffusing capacity in smokers has been previously shown to correlate with the presence of emphysema [2,3]. Two of these subjects had lung cancer. In the twenty-two patients without a significant smoking history, no subject had evidence of emphysema. Two subjects had bronchiectasis, one had pulmonary fibrosis and two subjects had asthma.

The primary indications for performing the bronchoscopies on the subjects were for assessment of: chronic cough (twelve subjects), airway infection (eight subjects), haemoptysis (seven subjects), possible interstitial lung disease (three subjects), exclusion of malignancy (two subjects), investigation of possible foreign body (one subject) and chest pain (one subject). Informed written consent was obtained from subjects prior to the bronchoscopy. Details of the subjects are listed in Table E1.

Bronchoscopy was performed in the bronchoscopy suite at MMC/Monash Health. BAL was performed as previously described [4] with multiple warmed aliquots of 25 ml of normal saline injected into the right middle lobe and aspirated.

Processing of the BAL

Approximately 10 ml of the returned fluid was sent to the Cytology Department in the Pathology Laboratories of Monash Health where differential count for white cell subtypes was performed. The remainder of the BAL was taken to the laboratories of the Department of Medicine, MMC. Samples were then spun down and washed twice, then re-suspended in culture media (Roswell Park Memorial Institute Medium (RPMI) -1640 (Sigma-Aldrich, St

Louis, MO), supplemented with 10% fetal calf serum, 0.1% L-glutamine (Sigma-Aldrich) and antibiotics (1% Penicillin (Sigma-Aldrich), 1% Streptomycin (Sigma-Aldrich) and 180 μg/ml Metronidazole (Claris, Chachamadi, Ahmedabad, India)). Cells were counted with a haemocytometer with trypan blue exclusion to obtain a viable cell count. Ammonium chloride (0.08%) was used to lyse red blood cells. More than 90% of the cells were viable with a mean macrophage count of 2.8×10^6 per subject. For confocal imaging, macrophages were seeded onto coverslips in 24-well flat-bottomed plates at a concentration of 1 to 3×10^5 cells per well and rested overnight.

Neutrophils

Peripheral blood neutrophils were obtained from healthy donors (the authors and laboratory staff) with informed consent prior to sample being taken. Neutrophils were isolated by density gradient using Polymorphprep (Progen Biotechnik, Heidelberg, Germany) [4] and seeded onto poly-l-lysine (Sigma-Aldrich) coated coverslips in 24-well plates for adhesion.

Cigarette smoke extract

An established method was used for the generation of cigarette smoke extract (CSE) for the *in vitro* experiments [5]. Cigarettes of the following composition were used: 16 mg of tar, 1.2 mg of nicotine, and 15 mg of CO_2. Smoke was generated in 50 ml tidal volumes over 10 seconds by use of a timed draw-back mimicking normal smoking inhalation volume and cigarette burn rate into media. One cigarette was used per 40 ml of media. Based on our previous work and preliminary experiments, undiluted CSE media was used with a one-hour incubation period. Preliminary experiments using both trypan blue exclusion and propidium iodide excluion, showed no significant differences in cell viability between control and CSE-exposed macrophages and neutrophils.

Smoking mouse model

To study the *in vivo* effect of cigarette smoke exposure an established smoking-mouse model was used [6]. This was a whole-body smoke exposure model. Male BALB-C mice (Animal Resource Centre, Perth, Australia), 6-8 weeks old were smoked three times a day for 4 days; with three cigarettes (each time) per 18 liter perspex enclosure (using same cigarettes that were listed above in the CSE section). Sham mice were only exposed to normal air. On the 4th day, BAL samples were taken (3 hrs after exposure to cigarette smoke) or for analysis of whole lung samples, lung tissue was inflated and fixed in 10% neutral formalin. To assess the effect of DNase, 1×10^4 international units per kg of deoxyribonculease (DNase) 1 (Dornase alfa, Roche Genentech, San Francisco, CA) was administered twice a day intraperitoneally. As a vehicle control for DNase group, mice were smoked and were administered with solution containing 0.15 mg/ml calcium chloride dehydrate and 8.77mg/ml sodium chloride at pH 6.3 (i.e. dornase alfa with DNase not present) at the same volume as DNase. Preliminary experiments using both trypan blue exclusion and propidium iodide excluion, showed no significant differences in cell viability between control and DNase-exposed macrophages and neutrophils. The doses of DNase used (both for *in vitro* and *in vivo* work) were based on our previous studies[4, 10].

Flow cytometry

We used a well-established flow cytometry method to measure the production of ROS from lung phagocytic cells [4,7]. The production of ROS causes cleavage of the dye dihydrorhodamine 123 (DHR), (Life Technologies, Carlsbad, CA), which leads to an increase in intracellular fluorescence measured on a Becton Dickinson (BD) FACS Canto II (BD Biosciences, San Jose, CA). Neutrophils or macrophages were either left untreated or were incubated with undiluted CSE for 1 h. DHR was added for the last 30 min and propidium

iodide (PI) was added immediately before flow cytometric analysis. Lung phagocytic cells (both macrophages and neutrophils) were gated, based on forward scatter/side scatter plot, PI negative population was selected and average mean fluorescence intensity of DHR was measured using FlowJo V. 10.0.8 software.

The cell impermeable DNA dye, SYTOX green (Thermofisher Scientific) and flow cytometric analysis was used to detect NETs from neutrophils. Neutrophils obtained from peripheral blood were stimulated with CSE or CSE/DNase or left untreated for 1 h. Neutrophils were stained with fixable viability dye eFluor 780 (eBioscience) for 30 min on ice, washed and resuspended in phosphate buffered saline (PBS). SYTOX green was added immediately before cytometric analysis. NET producing population was defined by firstly gating on viable cells from a forward scatter/side scatter plot then applying quadrant gating on SYTOX green and eFluor 780 dot plot. The NET forming population was designated as SYTOX green positive and negative for eFluor 780. This dye binds to free amine after penetrating the impaired cell membrane of dead cells. All flow cytometry analyses were performed on BD FACS Canto II (BD Biosciences, San Jose, CA) and data was analysed using FlowJo V.10.0.8 software (Tree Star, Ashland, OR).

Confocal Microscopy

Previously described methods were used [4,8-10]. BAL macrophages (1 to 3 × 10^5 per sample) or peripheral blood neutrophils from healthy donors were seeded (4 × 10^5 per sample) on poly-L-lysine coated coverslips and incubated for one hour 1), untreated 2), with CSE 3), with CSE and DNase (Roche Genentech) at 2 µg/ml. For these three experimental conditions, we also had 1), control-background (with secondary antibody only) and 2), isotype control with primary antibodies.

Staining of lung sections was performed on formalin-fixed, paraffin-embedded tissue specimens cut at 4 μm using Microtome and mounted on superfrost plus slides (Menzel, Braunschweig, Germany) as previously described by O'Sullivan KM et al [10]. Sections were dewaxed, rehydrated and pretreated with antigen retrieval solution Tris-EDTA pH 9 in a pressure cooker for 10 min, blocked (30 min) in 10% chicken sera in 5% bovine serum albumin (BSA) in PBS and probed with antibodies to define METs in macrophages against MMP-9, HCit3 and F4/80. To define NETs by neutrophils, sections were probed against neutrophil elastase, HCit3 and PAD4 antibodies, in 1% bovine serum albumin/PBS for 16 h at 4°C. Fluorescent detection was achieved by incubation with corresponding secondary antibodies, Alexa Fluor 488, 594, 647 as outlined in Supplementary Table E2 for 40 min at room temperature. Sections were washed in PBS and mounted with DAPI prolong gold (Molecular Probes). Fluorescent images were obtained using NIKON C1 confocal laser scanning head attached to Nikon Ti-E inverted microscope (Coherant Scientific, SA, Australia). The preparation was excited with 405, 488, 561 and 647 nm lasers. Single plane 512 x 512-pixel images were captured in a line-sequential manner (2 line averaging) using 20x 0.1N.A air and 40x 1.0N.A oil objectives. Ten fields of view per section were obtained for analysis and data for each result (i.e. 10 high power fields for control, 10 for smoke etc. for each mouse).

For immunofluorescence staining (for macrophages and neutrophils), cells were fixed in 2% periodate-lysine- paraformaldehyde (PLP) for 10 minutes, washed in PBS, permeabilized with 0.2% Tween 20 (Sigma-Aldrich) in PBS for 20 min followed by blocking with 10% chicken sera in 5% BSA diluted in PBS for 30 min. Primary and isotype control antibodies were incubated for 1 h at room temperature at 1:100 concentrations. The primary antibodies used for human samples were; sheep anti-human NE (LifeSpan Bioscience, Seattle, WA.),

mouse anti-human MMP9 (Novus Biological, Littleton, CO), rabbit anti-human MMP12 (Novus Biological), rabbit anti-human PADI2/PAD2 (Abcam, Cambridge, U.K.), mouse anti-human PADI4/PAD4 (Abcam) and rabbit anti-human H3Cit (Abcam). The primary antibodies used for mouse samples were goat anti-mouse MMP9 (Abcam) and rabbit anti-mouse Histone H3 (Abcam). Isotype control antibodies used were: Rat IgG2a, Mouse IgG1 (BD Biosciences), Mouse IgG2a (BioLegend), Rabbit IgG, Goat IgG and sheep IgG were purified in house using Protein G columns from sera. Cells were washed in PBS, and further incubated with the corresponding secondary antibodies (chicken anti-rabbit IgG AF 488, chicken anti-mouse AF 488, chicken anti-rabbit AF 594, chicken anti-rabbit AF 647, donkey anti-sheep AF 594, donkey anti-sheep AF 647 and chicken anti-goat AF 594 (all secondary antibodies were obtained from Life Technologies and were used at 1:400 dilution)) for 40 min at room temperature (antibodies used are listed in Table E2). Sections were washed in PBS and mounted with DAPI prolong gold for visualisation using NIKON C1 confocal microscope using the excitation 405, 488, 561 and 647 nm lasers and a 40x 1.0N.A oil objective.

In situ zymography was used to measure the area of the lung with proteolysis [11,12]. We used noninflated, nonfixed tissue for this method. Frozen lung sections of 4 µm were mounted on superfrost slides and pre-warmed in 1x reaction buffer for 5 minutes prior to the addition of fluorescein-labelled gelatin substrate (EnzChek Gelatinase/Collagenase Assay Kit; Molecular Probes). Fluorescent gelatin substrate, 30 µg/ml was directly added per section on individual slides. Slides were kept in a horizontal position and incubated in a light-protected humidified chamber at 37°C for 1 h. Sections were rinsed in 1x reaction buffer before mounted in 1x reaction buffer. As a negative control, sections received reaction buffer only, without fluorescent gelatin added. Lysis of the substrate was assessed by examination under Olympus

FV1200 inverted confocal microscope equipped 405 nm, 440 nm, 473nm, 543 nm and 635 nm lasers using the 60x 1.35N.A oil objective at a resolution of 512 x 512 pixels.

Microscopes were chosen on availability but all samples for the same experiments were done on the same microscope.

Image Analysis

Analysis was performed using IMARIS imaging analysis software (Bitplane AG, Zurich, Switzerland) and Image J. Cells extruding extracellular traps were determined based on extracellular chromatin detected by DAPI with coexpression of other markers (MMP9, MMP12, H3Cit, PAD2 PAD4, NE, F4/80)). For the human samples, comparison of MET/NET expression (between control, smoke and smoke/DNase treated groups) analysis was carried out on a minimum of 100 cells per sample (i.e. at least 100 cells were analysed for each control sample, smoke sample etc.) and cells were excluded from analysis if their staining was not above background/isotype control. For the mouse BAL samples, as the number of extracellular traps was lower, analysis was carried out on a minimum of 200 cells per sample. For the lung tissue samples, a minimum of 10 high power fields of view (FOV) were taken from each mouse for each stimulation (i.e. from each mouse; 10 FOV for control, 10 FOV for smoke, 10 FOV for smoke/DNase then results were averaged for each sample).

To determine the area of lung with evidence of proteolysis we used Image J to measure the area of lung that had staining above the background (this was done on minimum of 10 high power FOV for each sample) of the quenched sample and as another control, in lung tissue in which fluorescent gelatin had not been added.

The length of extracellular traps of macrophages and neutrophils was measured in ImageJ (National Institutes of Health, Bethesda, MD) based on extracellular chromatin. METS were identified by colocalisation of chromatin with MMP9/MMP12 and NETs by colocalisation of chromatin with NE, H3Cit and PAD4. The measurements of the extracellular trap lengths for each subject (minimum of 10 cells) were averaged to give the results in the text.

Statistical analysis

Statistical analysis was performed using Prism 6 software (GraphPad Software, San Diego, CA). Comparison between control and stimulated groups was done using paired or unpaired-testing with parametric or non-parametric methods as appropriate. Paired and unpaired t testing was used for parametric data (2 tailed). For non-parametric data; the Wilcoxon matched-pairs rank test was used for paired data and for unpaired data the Mann-Whitney test was used. A P value of less than 0·05 was considered to indicate statistical significance. Parametric results were represented graphically by mean and standard error of the mean, whilst non-parametric results were represented graphically by medians.

Table S1. Bronchoalveolar lavage (BAL) patient characteristics

	**Whole group**	**Non-smokers**	**Smokers**
**Number**	34	22	12
**Age (yrs.)**	56 ± 14	54 ± 16	61 ± 10
**Sex (male/female)**	21/13	12/10	9/3
**Smoking**			
**Pack years**	0 (0 - 30)	0 (0 – 0)	30 (21 - 48)
**Current smokers**	3	0	3
**Lung function (% predicted)**			
**FEV^{1}**	94 (86 – 100)	94 (85-98)	93 (87 -101)
**FVC**	98 (85 – 103)	97 (84 – 102)	102 (88 – 106)
**FER**	79 (73 – 85)	80 (76 – 85)	74 (62 – 79)
**DLCO**	82 (69 -94)	87 (80 – 96)	61 (46 – 70)

Footnote: Non-smokers = < 10 pack-years of smoking, smokers = > 10 pack-years of smoking, FEV^{1} = forced expiratory volume in 1 second, FVC = forced vital capacity, FER = forced expiratory capacity, DLCO = lung diffusing capacity for carbon monoxide. Results are expressed as mean ± standard deviation or median and interquartile range.

Table S2: Antibodies used for confocal microscopy

Human Studies

Primary Antibodies	Secondary Antibodies (All Life Technologies)	Supplier (1° AB)	Isotype Control
Rabbit anti-human H3Cit (Citrulline R26)	Chicken anti-rabbit AF 488/ Chicken anti-rabbit AF 594	Abcam	Rabbit IgG (In-house)
Rabbit anti-human MMP12	Chicken anti-rabbit AF 594	Novus Biological	Rabbit IgG (In-house)
Mouse anti-human MMP9	Chicken anti-mouse AF488	Novus Biological	Mouse IgG1 (BD Biosciences)
Rabbit anti-human PADI2/PAD2	Chicken anti-rabbit AF 594	Abcam	Rabbit IgG (In-house)
Mouse anti-human PADI4/PAD4	Chicken anti-mouse AF 647	Abcam	Mouse IgG2a (BioLegend)
Sheep anti-human NE	Donkey anti-sheep AF 594	LifeSpan Bioscience	Sheep IgG (In-house)

Mouse BAL/Lung tissue Studies

Primary Antibodies	Secondary Antibodies	Supplier (1^oAB)	Isotype Control
Goat anti-mouse MMP9	Chicken anti-goat AF 594	Abcam	Goat IgG (In-house)
Rabbit anti-mouse H3Cit (Citrulline R26)	Chicken anti-rabbit AF 488	Abcam	Rabbit IgG (In-house)
Rat anti-mouse F4/80	Chicken anti-goat AF 594	In-house (hybridoma)	Rat IgG2a (BD Biosciences)
Sheep anti-human Anti-HNE / NE	Donkey anti-sheep AF 647	Sapphire Bioscience	Sheep IgG (In-house)
Mouse anti-human PADI4/PAD4	Chicken anti-mouse AF 488.	Abcam	Mouse IgG2a (BioLegend)

Footnote: 1° AB = primary antibody, AF = Alexa Fluor, H3Cit = citrullinated histone, MMP = matrix metalloproteinase, PAD = peptidylarginase deiminase, NE = neutrophil elastase

Supplemental Figures

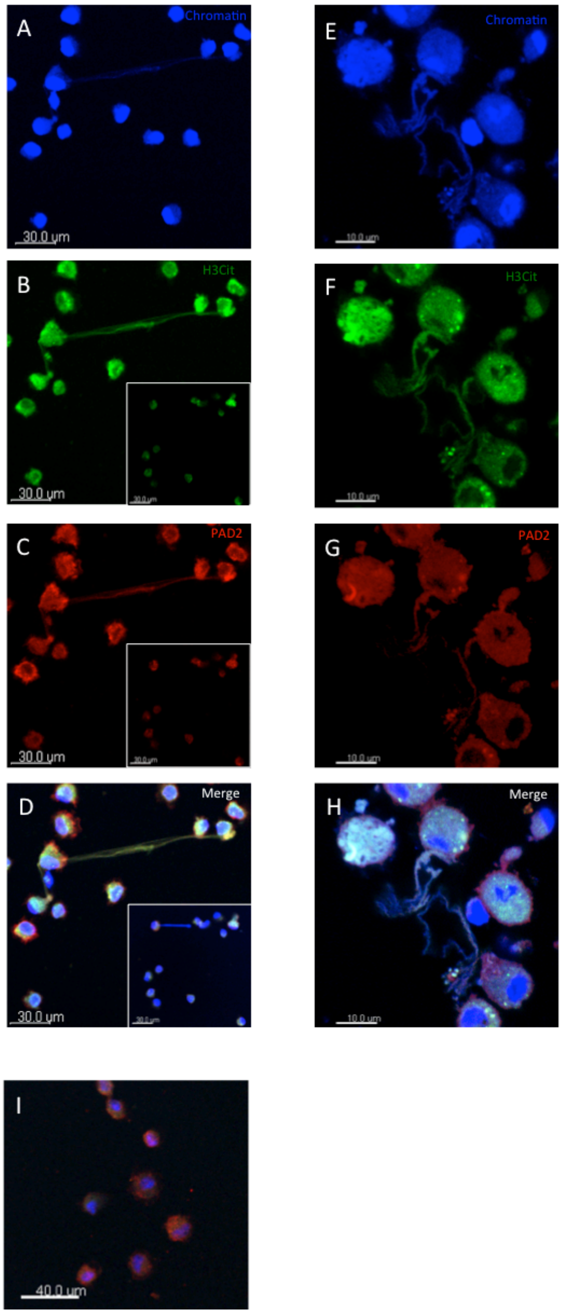

Figure S1. Human MET expression (H3Cit/PAD2). The figure shows an example of a MET made by a human alveolar macrophage exposed to cigarette smoke extract (CSE). **(A)** Shows staining for chromatin, **(B)** H3Cit, **(C)** PAD2, and **(D)** the merged image. Insert panel is isotype control. Scale bars = 30 µm. Panels **E** to **H** show higher magnification images (scale bars = 10 µm). Panel **I** shows macrophages exposed to culture medium without CSE (as control).

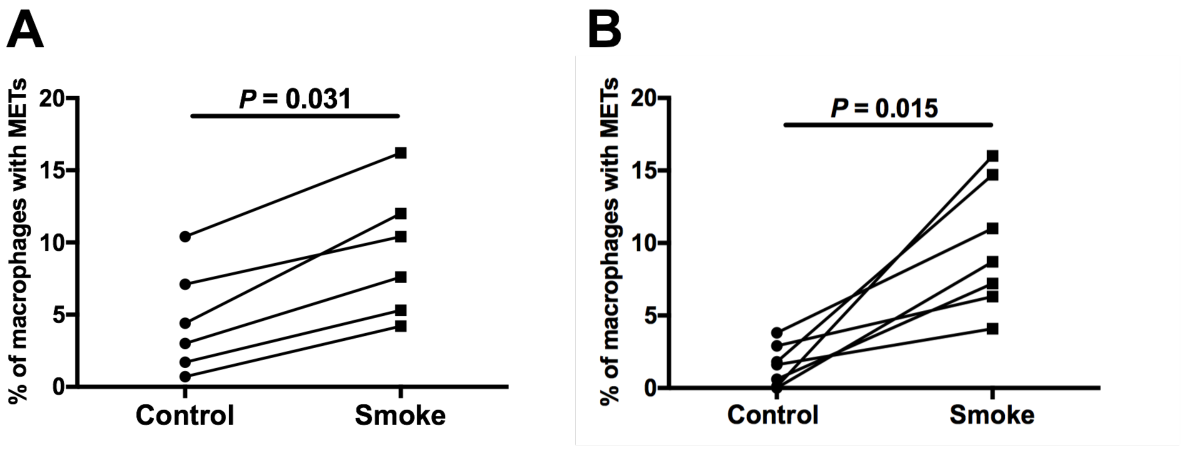

Figure S2. Human MET expression (H3Cit). The production of H3Cit is an essential step in the production of extracellular traps. We assessed the colocalisation of extracellular chromatin and H3Cit with either MMP9 or PAD2, when exposed to CSE in human BAL macrophages. **(A)** MET production was defined by colocalisation of extracellular chromatin, H3Cit and MMP9 (*n* = 6 individuals). **(B)** MET production was defined by colocalisation of extracellular chromatin, H3Cit and PAD2 (*n* = 7 individuals).

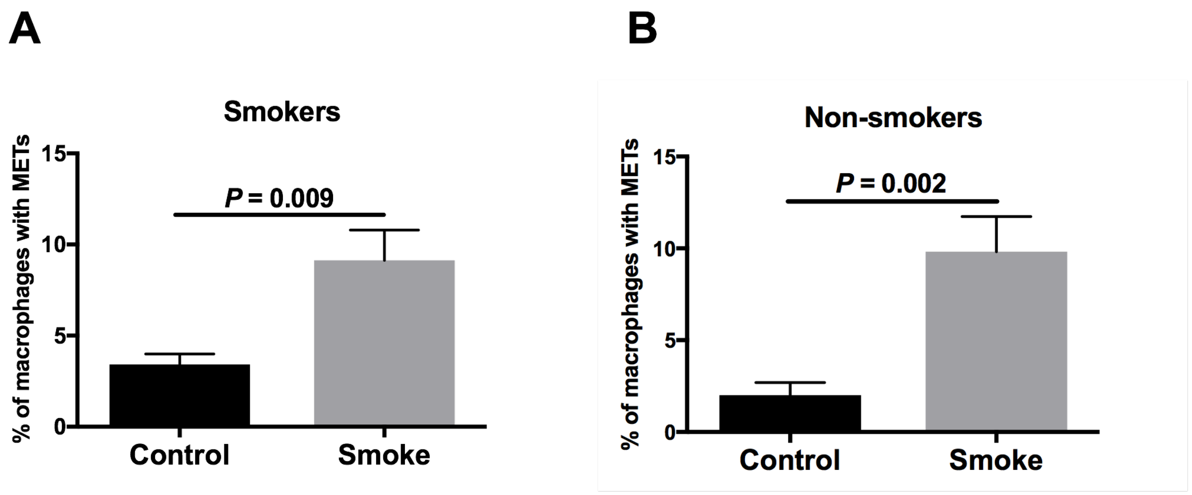

Figure S3. MET expression in smokers and non-smokers. MET expression by BAL macrophages in response to CSE exposure was measured in both smokers (with emphysema) and non-smokers (with no definable lung disease). MET production was defined by extracellular chromatin/MMP9/MMP12. CSE induced upregulation of MET expression in both groups (both groups, n = 8 individuals).

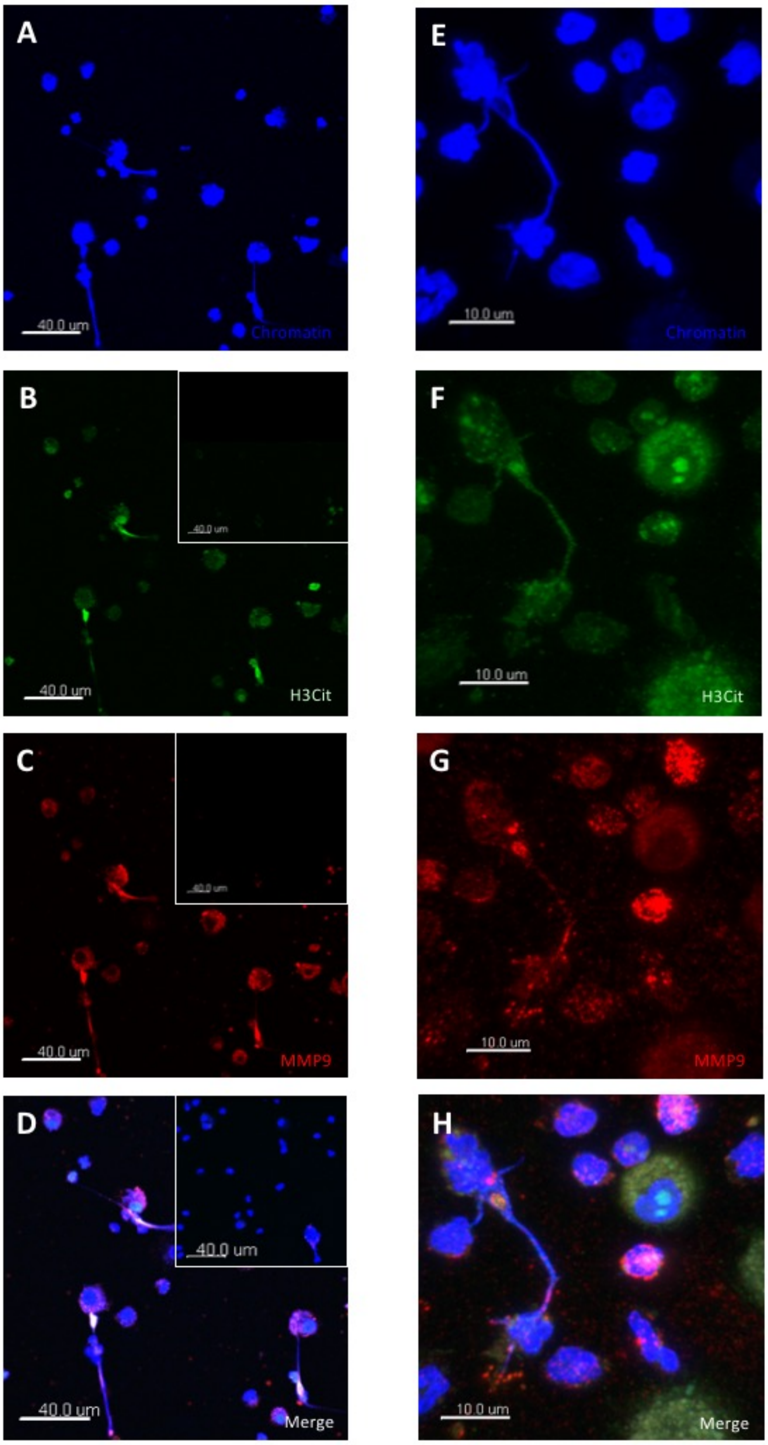

Figure S4. Mouse BAL MET expression. The figure shows an example of METs from *in vivo* smoke-exposed mouse BAL macrophages. **(A)** shows staining for chromatin, **(B)** H3Cit, **(C)** MMP9, and **(D)** the merged image. Insert panel is isotype control. Scale bars = 40 μm. Panels **E** to **H** show higher magnification images (scale bars = 10 μm).

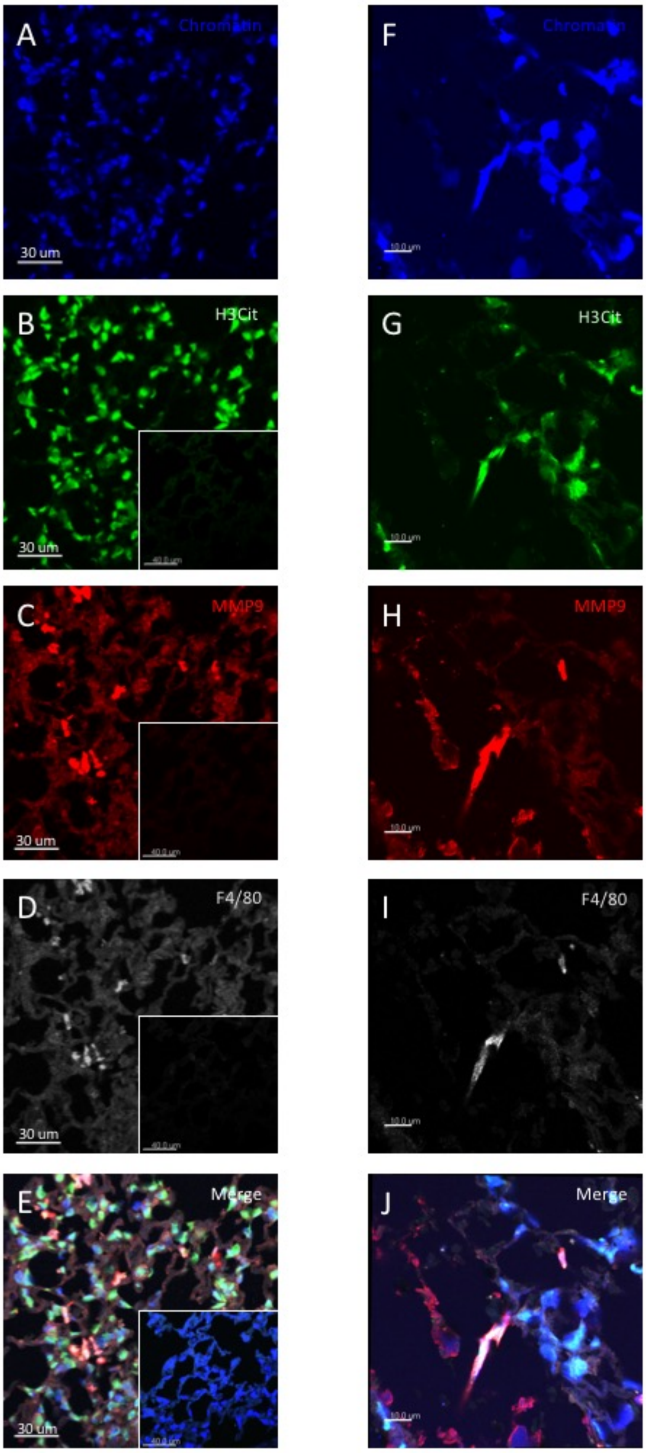

Figure S5. Mouse lung-tissue MET expression. The figure shows MET formation from *in vivo* smoke-exposed lung tissue. **(A)** Shows staining for chromatin, **(B)** H3Cit, **(C)** MMP9, **(D)** F4/80, and **(E)** the merged image. Insert panel is isotype control. Scale bars = 30 μm (40 μm for isotype). Panels **F** to **J** show higher magnification images (scale bars = 10 μm).

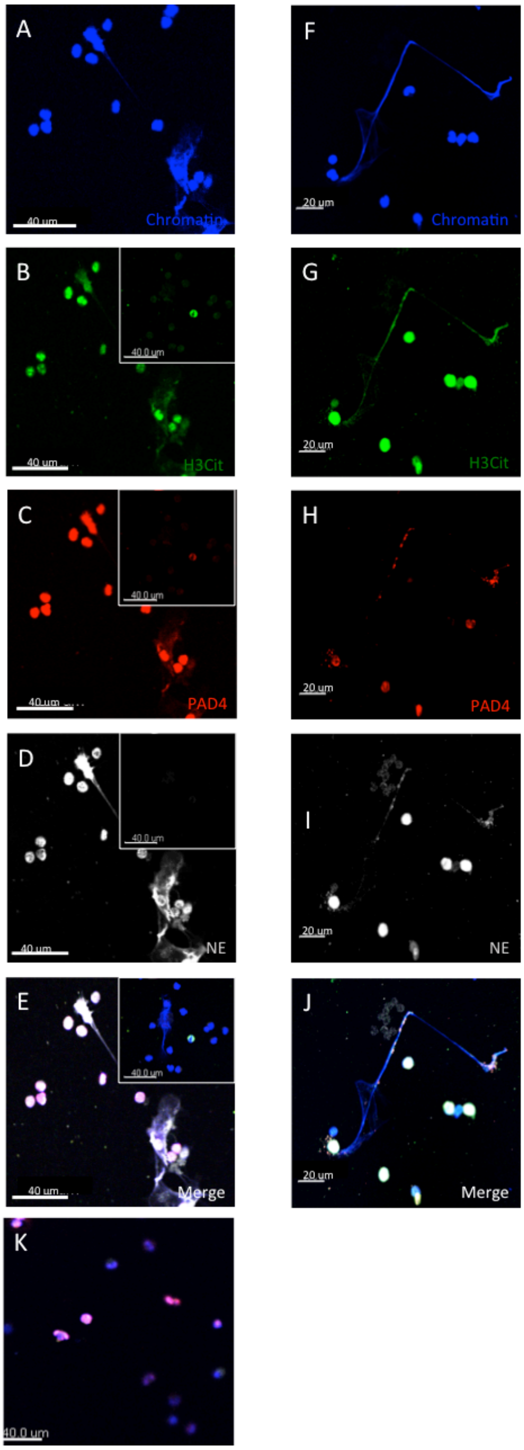

Figure S6. Human NET expression. The figure shows an example of NET formation in human blood neutrophil exposed to CSE. **(A)** Shows staining for chromatin, **(B)** H3Cit, **(C)** PAD4, **(D)** NE, and **(E)** the merged image. Insert panel is isotype control. Scale bars = 40 μm. Panels **F** to **J** show features of a NET formation in a single neutrophil (scale bars = 20 μm). Panel **K** shows neutrophils exposed to culture medium without CSE (as control).

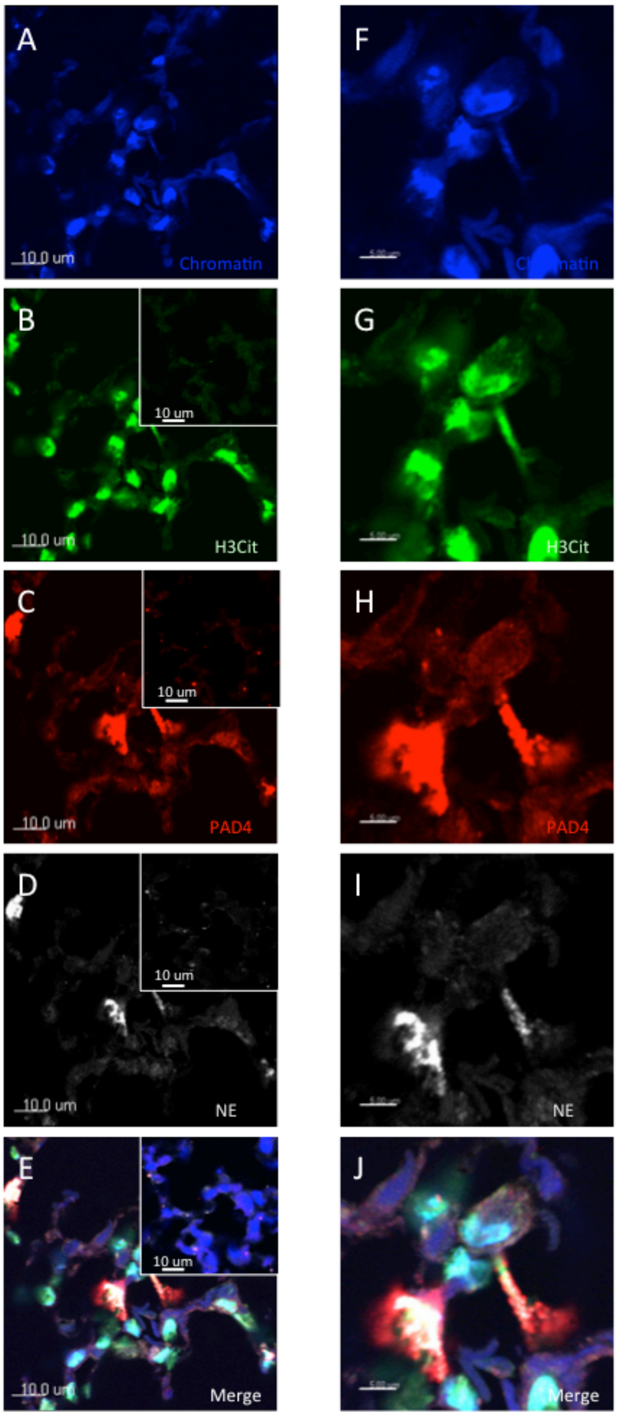

Figure S7. Mouse NET expression. The figure shows NET formation from *in vivo* smoke-exposed lung tissue. **(A)** Shows staining for chromatin, **(B)** H3Cit, **(C)** PAD4, **(D)** NE, and **(E)** the merged image. Insert panel is isotype control. Scale bars = 10 μm. Panels **F** to **J** show higher magnification images (scale bars = 5 μm).

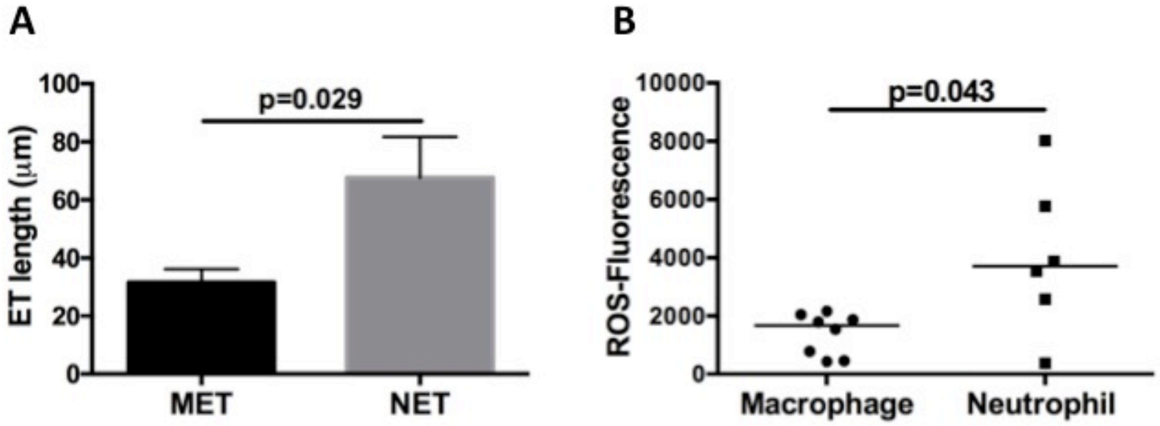

Figure S8. Comparison of METs/NETs and macrophages/neutrophils in human cells exposed to CSE *in vitro*. **(A)** Shows the morphology of the extracellular traps (ET) produced by the two cell types was different with NETs having a longer extension of extracellular chromatin (designated ET length). **(B)** ROS production was higher in the neutrophils.

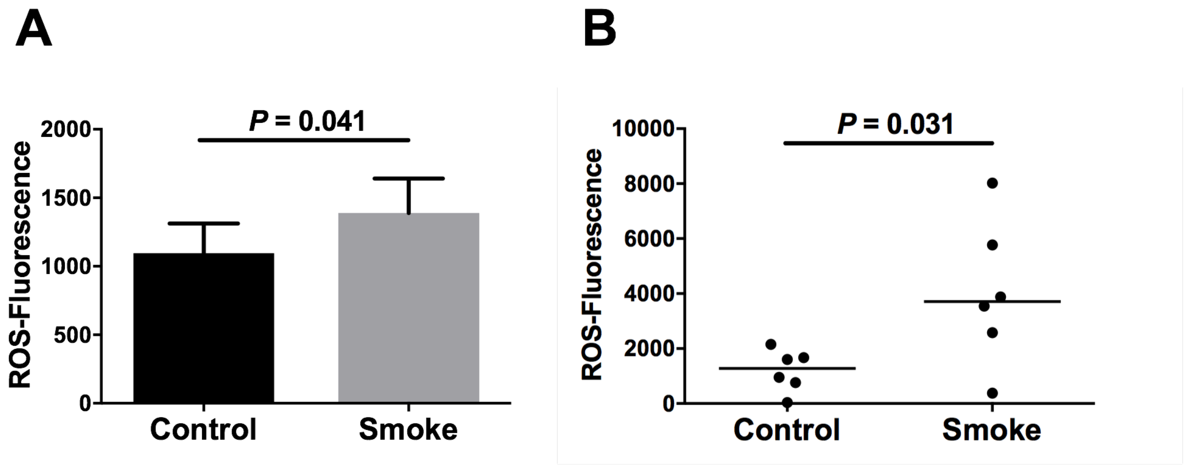

Figure S9. **CSE increases ROS production as measured by DHR cleavage-induced fluorescence, measured by flow cytometry.** **(A)** Increase in ROS in human BAL macrophages exposed to CSE (*n* = 8 individuals). **(B)** Increase in ROS in human neutrophils exposed to CSE (*n* = 6).

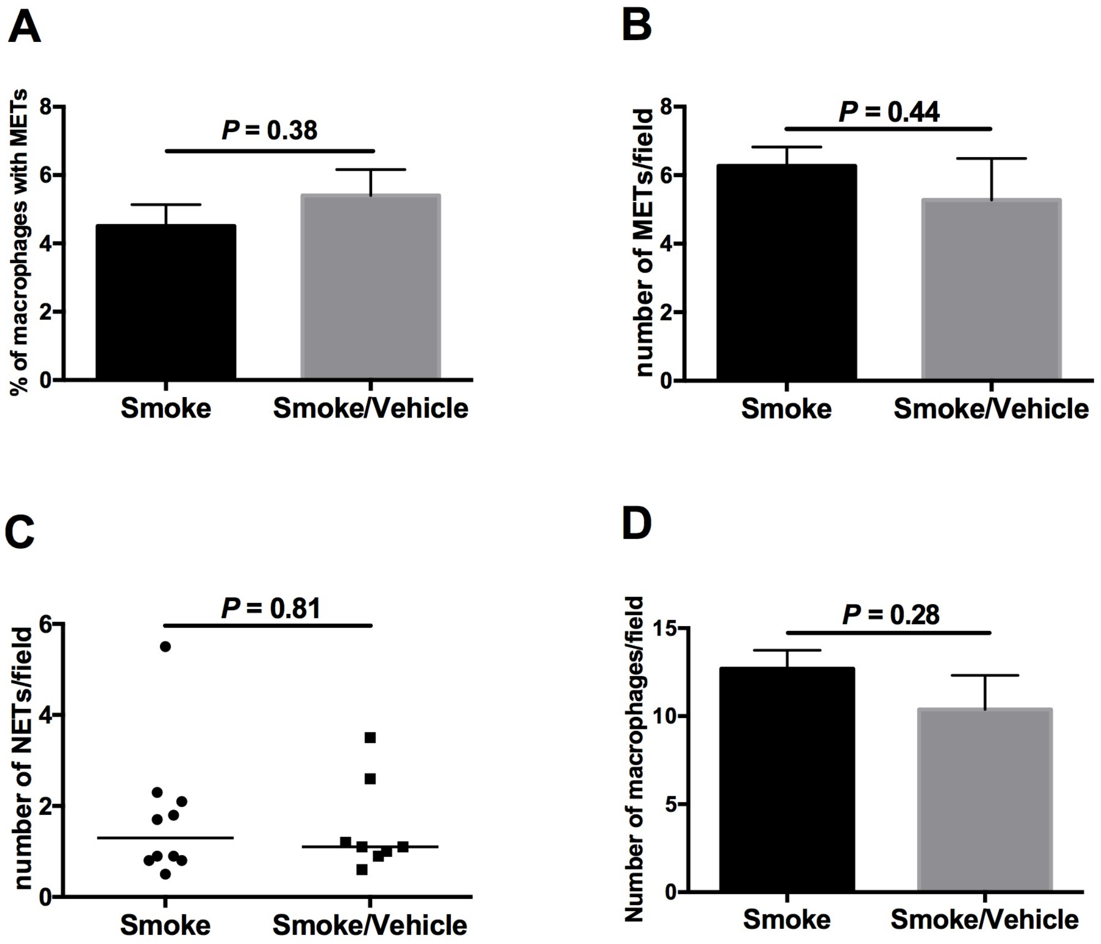

Figure S10. Effects of Smoke and DNase vehicle control. In our experiments, we have shown that DNase 1 reduces the expression of NETs/METs and lung macrophages after smoke exposure. As another form of control, we assessed the effect of the DNase vehicle on NET/MET formation and macrophage cells in BAL samples and in lung tissue from *in vivo* smoke-exposed mice. There were no significant reductions in **(A)** BAL MET expression, **(B)** lung MET expression, **(C)** lung NET expression, or **(D)** numbers of lung macrophages.

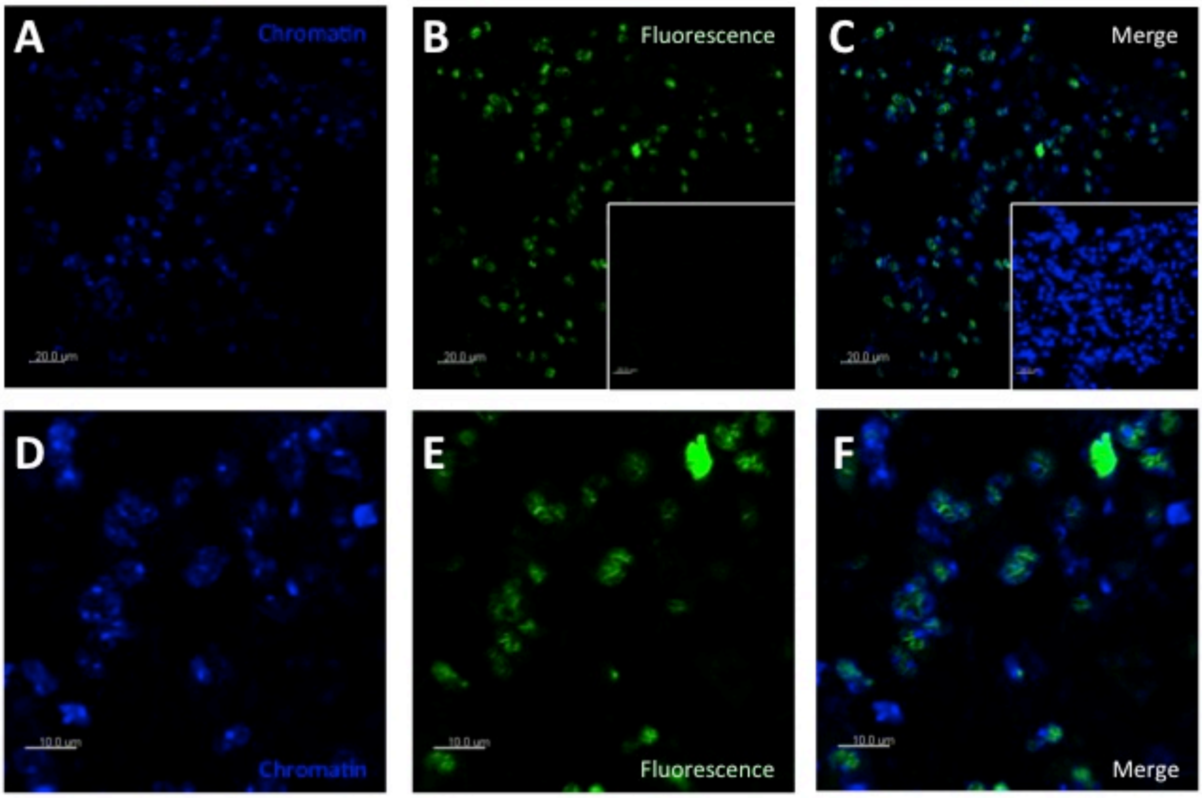

Figure S11. *In situ* zymography of lung tissue exposed to cigarette smoke. The area of the lung with proteolytic activity was measured by the presence of green fluorescence. This lung tissue was not fixed/inflated (unlike the previous samples). **(A)** Shows staining for chromatin, **(B)** quenched fluorescence as a marker of proteolysis, and **(C)** the merged image. Insert panel is control (without fluorescent gelatin added). Scale bars = 20 μm. Panels **D** to **F** show higher magnification images (scale bars = 10 μm).

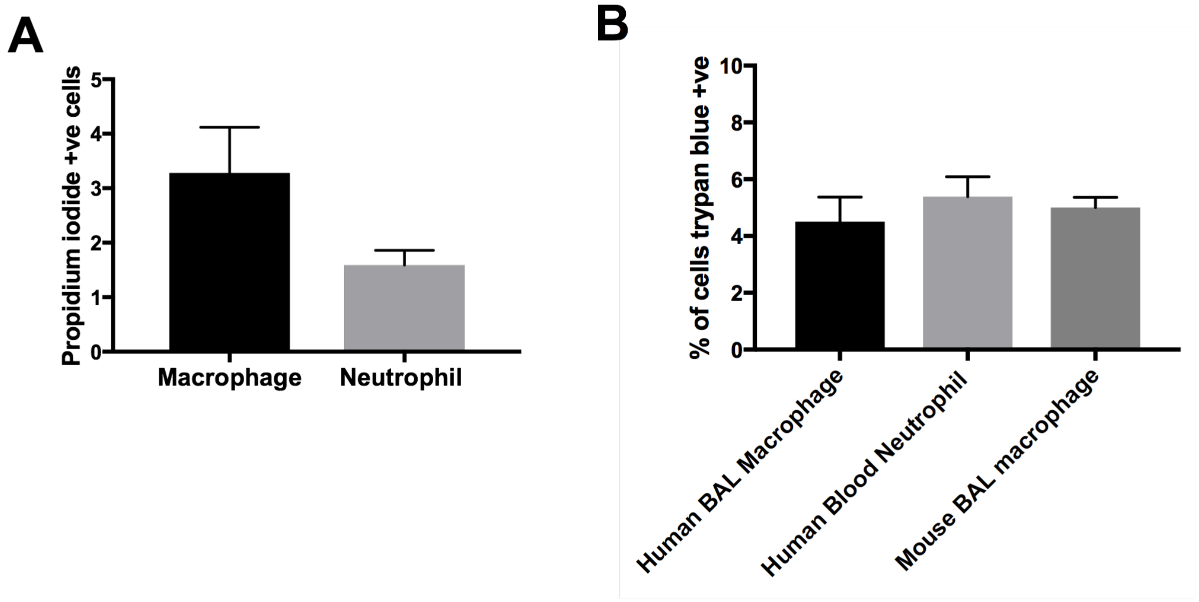

Figure S12. Preliminary experiments were undertaken to assess the effect of DNase on cell viability. (A) Shows the effect of the dose of DNase used for *in vitro* experiments on cell death as assessed by propidium iodide (PI) staining in macrophages (n = 6) and blood neutrophils (n = 6). Dead cells are +ve for PI and using this model there were on average less than 2-3% of dead cells with the dose of DNase used for the experiments. **(B)** Shows the effect of the dose of DNase used for *in vitro* experiments on cell death as assessed by positive staining for trypan blue staining in human macrophages (n = 8) and blood neutrophils (n = 8) and *in vivo* in mouse BAL macrophages (n = 6). Dead cells are +ve for trypan blue and using this model there were on average less than 4-5% of dead cells with the dose of DNase used for the experiments.

References

1 Vogelmeier, C. F. *et al.* Global Strategy for the Diagnosis, Management, and Prevention of Chronic Obstructive Lung Disease 2017 Report. GOLD Executive Summary. *Am J Respir Crit Care Med.* **195**, 557-582 (2017).

2 Morrison, N. J. *et al.* Comparison of single breath carbon monoxide diffusing capacity and pressure-volume curves in detecting emphysema. *Am Rev Respir Dis.* **139**, 1179-1187 (1989).

3 Gould, G. A. *et al.* Lung CT density correlates with measurements of airflow limitation and the diffusing capacity. *Eur Respir J.* **4**, 141-146 (1991).

4 King, P. T. *et al.* Nontypeable Haemophilus influenzae induces sustained lung oxidative stress and protease expression. *PloS one.* **10**, e0120371 (2015).

5 Ruwanpura, S. M. *et al.* Interleukin-6 promotes pulmonary emphysema associated with apoptosis in mice. *Am J Respir Cell Mol Biol.* **45**, 720-730 (2011).

6 Vlahos, R. *et al.* Differential protease, innate immunity, and NF-kappaB induction profiles during lung inflammation induced by subchronic cigarette smoke exposure in mice. *Am J Physiol. Lung Cell Mol Physiol.* **290**, L931-945 (2006).

7 King, P. T. *et al.* Assessing immune function in adult bronchiectasis. *Clin Exper Immunol.* **144**, 440-446 (2006).

8 Brinkmann, V. & Zychlinsky, A. Neutrophil extracellular traps: is immunity the second function of chromatin? *J Cell Biol.* **198**, 773-783 (2012).

9 Brinkmann, V. *et al.* Neutrophil extracellular traps kill bacteria. *Science.* **303**, 1532-1535, (2004).

10 O'Sullivan, K. M. *et al.* Renal participation of myeloperoxidase in antineutrophil cytoplasmic antibody (ANCA)-associated glomerulonephritis. *Kidney Int.* **88**, 1030-1046, (2015).

11 Vandooren, J., Geurts, N., Martens, E., Van den Steen, P. E. & Opdenakker, G. Zymography methods for visualizing hydrolytic enzymes. *Nat Methods.* **10**, 211-220 (2013).

12 Duran-Vilaregut, J. *et al.* Role of matrix metalloproteinase-9 (MMP-9) in striatal blood-brain barrier disruption in a 3-nitropropionic acid model of Huntington's disease. *Neuropathol Applied Neurobiol.* **37**, 525-537 (2011).
